# Supplementary material for: Universal principles of cell population growth follow from local contact inhibition
Source: iScience. 2026 May 15;29(6):115953. doi: 10.1016/j.isci.2026.115953 (PMC13207371; doi:10.1016/j.isci.2026.115953)
Supplement: Document S1. Figures S1–S4 [file mmc1.pdf]

iScience, Volume *29*

Supplemental information

Universal principles of cell population growth follow from local contact inhibition

Gregory J. Kimmel, Sadegh Marzban, Mehdi Damaghi, Arne Traulsen, Alexander R.A. Anderson, Jeffrey West, and Philipp M. Altrock

Supplementary Figures

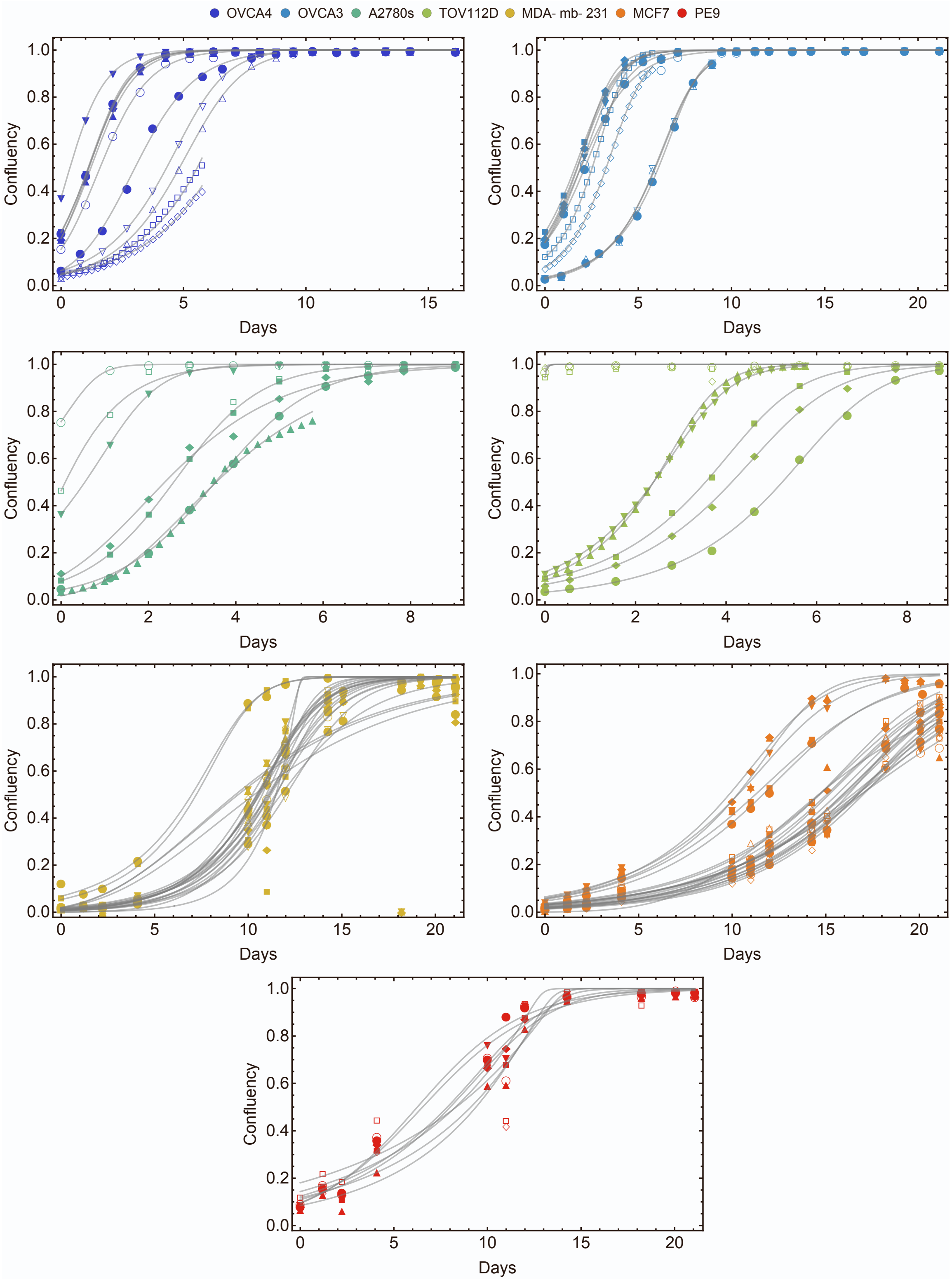

Figure S1. **Fitting a generalized logistic growth law to longitudinal *in vitro* growth data of the seven cell lines (see Methods).**

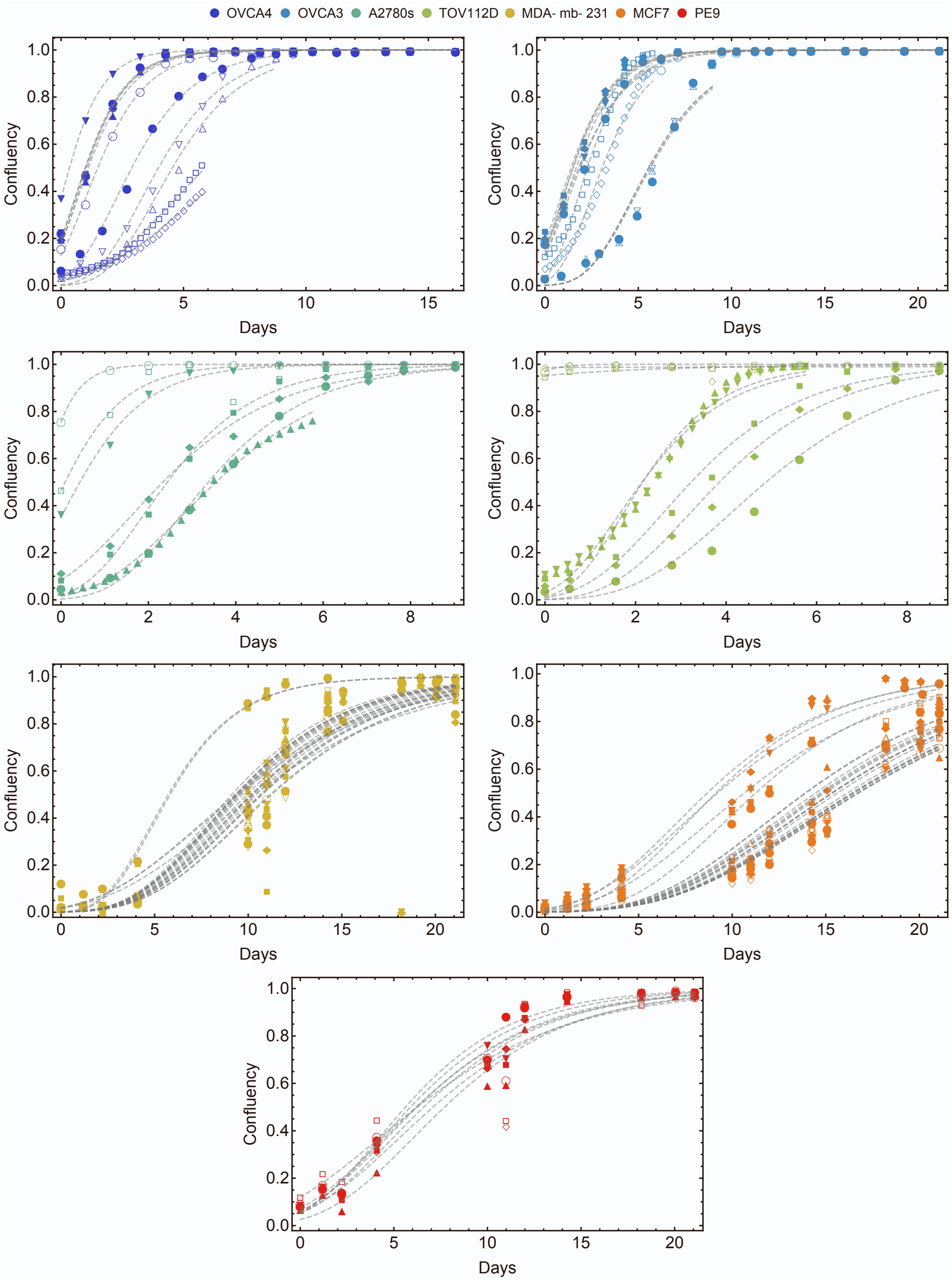

Figure S2. **Fitting a Gompertz growth law to longitudinal** ***in vitro*** **growth data of the seven cell lines (see Methods).**

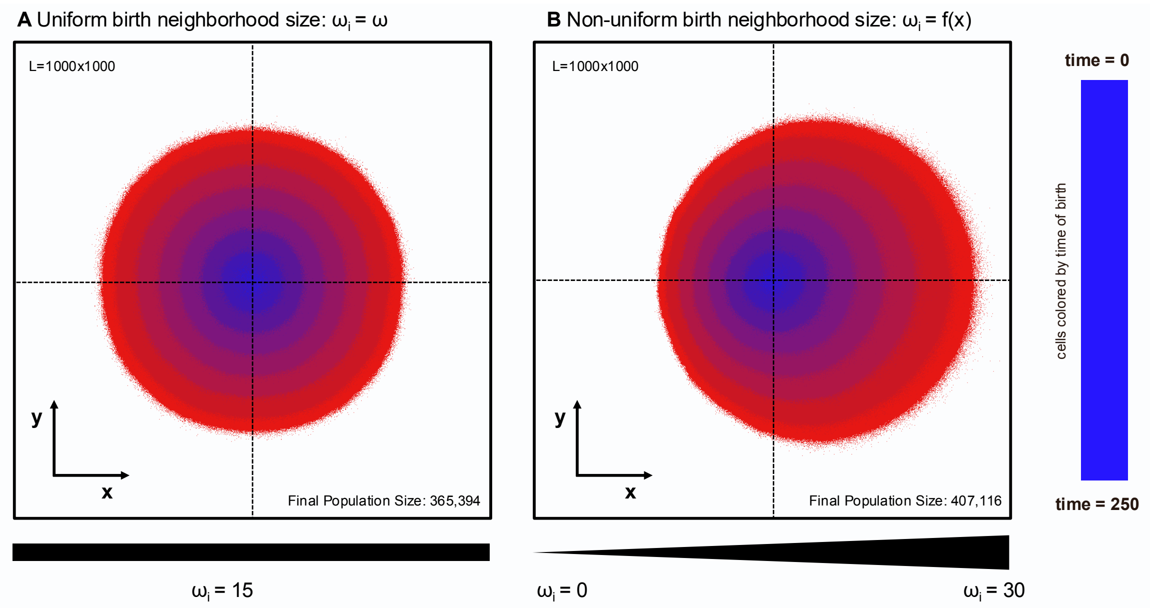

Figure S3. Uniform versus non-uniform birth neighborhood size in space. (A) A single stochastic simulation ($l = 10^6$; $b = 0.1$) with a constant birth neighborhood size ($\omega_i = 15$) seeded with a single cell at the center of the domain. (B) An identically parameterized stochastic simulation as in (A), but with non-uniform, varying birth neighborhood size. On the left-hand side of the domain $\omega_i = 0$, while increasing linearly such that the right-hand side $\omega_i = 30$. Despite having the same average neighborhood size throughout the domain, the non-uniform simulation grows to a larger population size, and is skewed toward the right-hand side. Dashed lines indicate the domain center, to guide the eye.

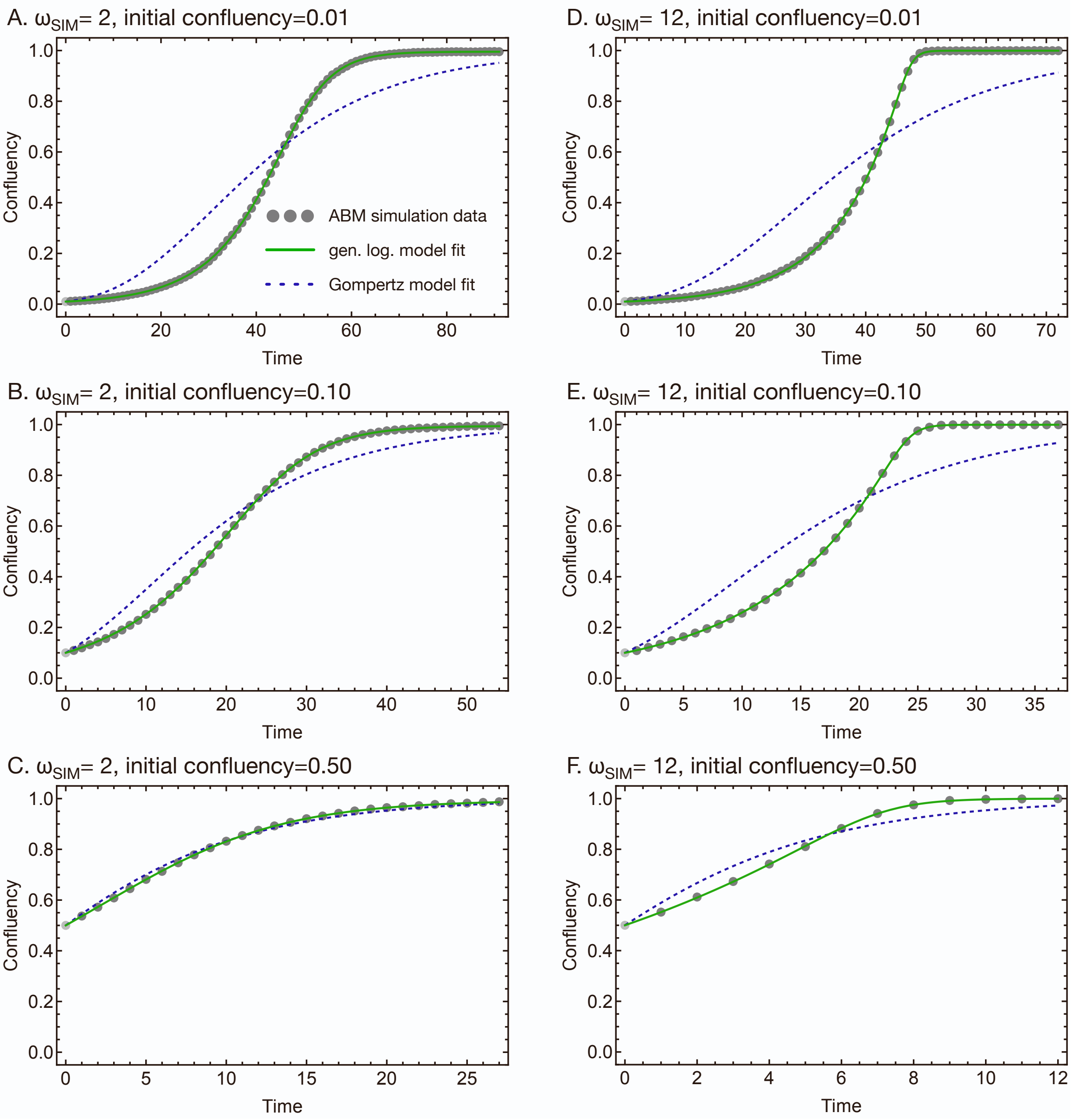

Figure S4. Example fits of generalized logistic (gen. log.) and Gompertz models to agent-based model simulation data. Generalized logistic model (solid lines) and Gompertz model (dashed lines) fits do different simulated agent-based (ABM) models, across different initial conditions (confluencies) and for different implemented birth-neighborhood values ω_{SIM}.
